# Koolen‐de Vries syndrome in a 63‐year‐old woman: Report of the oldest patient and a review of the adult phenotype

**DOI:** 10.1002/ajmg.a.62536

**Published:** 2021-10-19

**Authors:** Marianna Farnè, Laura Bernardini, Anna Capalbo, Giusy Cavarretta, Barbara Torres, Mariabeatrice Sanchini, Sergio Fini, Alessandra Ferlini, Stefania Bigoni

**Affiliations:** ^1^ Medical Genetics Unit, Department of Medical Sciences University of Ferrara Ferrara Italy; ^2^ Medical Genetics Unit IRCCS Casa Sollievo della Sofferenza Foundation San Giovanni Rotondo (FG) Italy; ^3^ Medical Genetics Unit, Department of Mother and Child Ferrara Sant'Anna University Hospital Ferrara Italy

**Keywords:** 17q21.31 microdeletion, adult phenotype, *KANSL1*, Koolen‐de Vries syndrome

## Abstract

Koolen‐de Vries syndrome (KdVS) is a rare genetic disorder caused by a de novo microdeletion in chromosomal region 17q21.31 encompassing *KANSL1* or by a de novo intragenic pathogenic variant of *KANSL1*. KdVS is typically characterized by intellectual disability (ID), variable from mild to severe, developmental psychomotor delay, especially of expressive language development, friendly disposition, and multiple systemic abnormalities. So far, most of the individuals affected by KdVS are diagnosed in infancy or in adolescence; to the best of our knowledge, only 34 (including ours) adults have been reported in literature. Here we present the adult phenotype of a 63‐year‐old Italian woman affected by KdVS, caused by a 17q21.31 microdeletion. She is, to our knowledge, the oldest affected individual reported so far. We collected her clinical history and photographs, as well as those of other 26 adult patients described so far and compared her to them. We propose that the cardinal features of KdVS in adulthood are ID (ranging from mild to severe, usually moderate), friendly behavior, musculoskeletal abnormalities (especially scoliosis), and facial dysmorphism (a long face and a pronounced pear‐shape nose with bulbous overhanging nasal tip). Therefore, we suggest considering KdVS in differential diagnosis in adult patients characterized by these features.

## 1 INTRODUCTION

Koolen‐de Vries syndrome (KdVS), also known as 17q21.31 microdeletion syndrome or *KANSL1*‐related intellectual disability (ID) syndrome (OMIM #610443), is a rare genetic disorder described for the first time in 2006 (Koolen et al., 2006). It was initially described to be caused by a ~400‐kb to ~650‐kb microdeletion in chromosomal region 17q21.31 encompassing at least six genes, among them *KANSL1* (OMIM*612452) (Koolen et al., 2008; Sharp et al., 2006; Shaw‐Smith et al., 2006). In 2012, it was discovered that de novo intragenic heterozygous variants of *KANSL1*, leading to haploinsufficiency of the gene, are sufficient to cause the same phenotype of a 17q21.31 microdeletion. Therefore, it was evident that KdVS can be caused by either a 17q21.31 microdeletion encompassing *KANSL1* or a heterozygous intragenic pathogenic variant in *KANSL1* gene (Koolen, Dupont, et al., 2012; Zollino et al., 2012). Genotype–phenotype correlation studies did not show important clinical differences between the two groups of patients (Koolen et al., 2016; Zollino et al., 2015). *KANSL1* is a widely expressed gene and encodes the KAT8 regulatory NSL complex Subunit 1, which belongs to a complex involved in chromatin modification (Koolen, Dupont, et al., 2012).

The prevalence of KdVS was initially estimated as one in 16,000 individuals (Koolen et al., 2008). Following reports of deletions involving *KANSL1* in a cohort of children with developmental delay and/or ID showed a frequency of 0.11% and a prevalence of 1 in 55,000 individuals (Koolen et al., 2016). The prevalence of the *KANSL1* SNVs is still to be assessed, due to limited number of known patients, but preliminary data suggest that they might be as frequent as deletions (Koolen et al., 2016).

KdVS is typically characterized by developmental psychomotor delay, which especially involves expressive language development, and ID, which can vary from mild to severe. Neonatal hypotonia is a common characteristic of KdVS and may be associated with feeding difficulties and hospitalization during the first year of life. IUGR and low birth weight can occur but body weight often improves; short stature, if present, is proportionate (Koolen et al., 2016; Zollino et al., 2015). Epilepsy is reported in about 50% of patients, with typical childhood‐onset focal seizures, multifocal epileptiform discharge EEG patterns and various structural brain abnormalities in MRIs (Myers et al., 2017). Individuals with KdVS are typically characterized by an amiable and friendly disposition comparable to Angelman and Williams Syndromes, with a strong memory for social‐contextual information (Egger et al., 2013). Behavioral problems such as ADHD, ASD, anxiety, and stammering have also been reported (Koolen et al., 2016). Facial dysmorphism is typically reported in KdVS patients, including a long face, upward slanting palpebral fissures, narrow/short palpebral fissures, ptosis, epicanthal folds, tubular or pear‐shaped nose, bulbous nasal tip, everted lower lip, and large/prominent ears (Koolen et al., 2016). Adult individuals with KdVS have been described to have a longer face, with a broadening of the chin and a more pronounced tubular or pear‐shaped nose (Koolen et al., 2008). Multiple systemic abnormalities have been described with a variable percentage involving the following body districts: musculoskeletal (tracheo/laryngomalacia, pectus excavatum or carinatum, scoliosis/kyphosis, hip dislocation/dysplasia, joint hypermobility and positional deformities of the feet), visual (hypermetropia, strabismus, cataract) and hearing (conductive/sensorineural hearing impairment), cardiovascular (mainly atrial and ventricular septal defects), various renal and urogenital anomalies, and different ectodermal abnormalities (Koolen et al., 2016; Zollino et al., 2015).

From a genetic standpoint, all cases reported so far are “de novo.” Parental molecular analysis of the 17q21.31 region has demonstrated that in all patients with all 17q21.31 microdeletion, at least one parent is a carrier of the H2 haplotype, which is associated with a common 900‐kb inversion polymorphism, present in ~20% of the European population (Koolen et al., 2008). Therefore, this inversion is a necessary (but not sufficient) factor for the deletion to occur (Koolen et al., 2008). Being caused by a de novo event, the risk of recurrence for parents with a child with KdVS is low. However, the possibility of a parental germinal mosaicism (at least two families already reported) or of a parental balanced chromosomal rearrangement involving 17q21.31 must be taken into consideration (Koolen, Kramer, et al., 2012). So far, most of the individuals affected by KdVS have been diagnosed in infancy or in adolescence (Ciaccio et al., 2016; Koolen et al., 2016; Zollino et al., 2015). Also fetal cases have been reported (Sauvestre et al., 2017).

Here we present an adult phenotype of a 63‐year‐old Italian woman affected by KdVS, who is, to our knowledge, the oldest affected individual reported so far in literature. Moreover, we searched for all the adult patients with KdVS in literature: we identified 34 (with our patient included) adult individuals, of whom 15 are males (14 with a 17q21.31 microdeletion, one with a *KANSL1* pathogenic variant) and 19 are females (16 with a 17q21.31 microdeletion, three with a *KANSL1* pathogenic variant) (Ciaccio et al., 2016; Dubourg et al., 2011; Koolen et al., 2006, 2008, 2016; Moreno‐Igoa et al., 2015; Morgan et al., 2018; Myers et al., 2017; Nascimento et al., 2017; Shaw‐Smith et al., 2006; Terrone et al., 2012; Zollino et al., 2015; lastly, while concepting this manuscript, Amenta et al., 2020 and Pascolini et al., 2021). In particular, we decided to collect the clinical characteristics of 27 (13 males and 14 females) out of 34 adult patients, excluding the seven patients (Morgan et al., 2018; Myers et al., 2017) whose clinical description was more focused on epileptology and on speech development respectively, in order to delineate the adult phenotype of this rare disease, which seems to be mainly characterized by ID, friendly behavior, musculoskeletal abnormalities, and facial dysmorphism.

## 2 CASE REPORT

### 2.1 Clinical evaluation

A 38‐year‐old secundigravida woman was admitted to our genetic counseling service for a prenatal genetic consultation in 2019 in order to define the recurrence risk of a moderate ID present in her then 61‐year‐old paternal aunt. We collected the aunt's personal and family history (with the help of her brother as her parents were dead) (Table 1, patient P1). Written informed consent for publication of both clinical data and photographs was obtained from the patient, with approval of her family.

**TABLE 1:** Clinical and molecular findings of 27 adult patients affected by Koolen‐de Vries syndrome reported in literature

	P1, present case	P2 (Koolen et al., 2008)	P3 (Dubourg et al., 2011)	P4 (Terrone et al., 2012)	P5 (Nascimento et al., 2017)	P6 (Moreno‐Igoa et al., 2015)	P7 (Amenta et al., 2020)	P8 (Amenta et al., 2020)	P9 (Koolen et al., 2008; Shaw‐Smith et al., 2006)
Gender	Female	Male	Male	Female	Female	Female	Female	Female	Male
Age, years	63	18	18	18	18	19	19	19	20
Genetics	17q21.31 microdeletion	17q21.31 microdeletion	17q21.31 microdeletion	17q21.31 microdeletion	17q21.31 microdeletion	Complex rearrangement^a^	17q21.31 microdeletion	17q21.31 microdeletion	17q21.31 microdeletion
Gestational age, weeks	>41	n.a.	40	at term	38	36	40	34	term
Birth weight, g	n.a.	Not low	2990 (10th)	2630 (5th–10th)	2550	1860	3150 (25th–50th)	2230 (50th–75th)	2700
Height, cm	143 (−3 *SD*)	n.a.	n.a.	n.a.	n.a.	At the 10th	158 (−1.2 *SD*)	150 (−2.2 *SD*)	<0.4th
Postnatal short stature	+	−	+	n.a.	n.a.	−	+	+	+
Weight, kg	60	n.a.	n.a.	n.a.	n.a.	At the 90th	63 (50th–75th)	53 (25th)	0.4th–2nd centile
BMI	29.3	n.a.	n.a.	n.a.	n.a.	n.a.	25.24	23	n.a.
*Neurological/neuropsychological features*									
Hypotonia	+	+	+	+	n.a.	+	+	+	+
Feeding problems	+	+	+	n.a.	n.a.	+	+	+	+
Intellectual disability	+ moderate	+ moderate	n.a.	+ moderate	+ mild	+	+ mild	+ moderate	+ moderate/severe
Intelligence quotient (IQ)	n.a.	n.a.	n.a.	50	n.a.	n.a.	53	40	n.a.
Seizures/EEG anomalies	−	+	+	+	+	n.a.	−	+	+ petit mal
Enlarged ventricles and hydrocephalus	n.a.	+	−	−	n.a.	+	−	−	−
Other structural CNS anomalies	n.a.	+	+ dysgenesis of the corpus callosum	+ mild enlargement of some perivascular spaces of Virchow‐Robin in the white matter	n.a.	+	−	+ mild hyperintensity of the periventricular white matter	n.a.
Friendly/amiable affect	+	+	n.a.	+	+	−	+	+	+
Stereotypic behavior	−	n.a.	n.a.	−	n.a.	+	−	−	n.a.
Anxiety	+	n.a.	n.a.	−	n.a.	n.a.	+	+	n.a.
Difficulty to perform ADL	+	n.a.	n.a.	+	n.a.	+	−	+	n.a.
Other	Stutterer, strong memory for social information	Agenesis of corpus callosum	Behavioral problems					Shyness, marked emotionality	
*Dysmorphic features*									
Long face	+	−	+	n.a.	+	+	+	n.a.	+
Upslanting palpebral fissures	+ in infancy	−	n.a.	+	+	+	−	n.a.	−
Ptosis	+/− (droopy eyelids)	−	n.a.	n.a.	+	+	+/−	n.a.	−
Epicanthal folds	−	−	n.a.	+	n.a.	+	−	n.a.	−
Tubular or pear‐shaped nose	+	+	+	+	+	+	+	n.a.	+
Everted lower lip	−	+	+	n.a.	−	−	−	n.a.	n.a.
Large/prominent ears	+	−	+	– (low‐set ears)	−	−	−	n.a.	−
Other	Triangle‐shaped helix of the right ear		High and broad forehead, gingival pads			Hypoplastic nares			Deep‐set eyes, high palate, broad chin
*Hearing and visual impairment*									
Hypermetropia	n.a.	−	n.a.	−	n.a.	−	−	−	+
Strabismus	−	−	n.a.	−	n.a.	−	−	−	−
Retinal impairment	−	n.a.	n.a.	−	n.a.	n.a.	−	−	+
Hearing impairment	+ (conductive, middle age onset)	n.a.	n.a.	−	n.a.	n.a.	−	−	n.a.
Other	Bilateral cataract (middle age onset)		Mild myopia			Iris heterochromia			
*Musculoskeletal anomalies*									
Tracheo/laryngomalacia	−	n.a.	n.a.	n.a.	n.a.	n.a.	−	+	n.a.
Pectus deformities	−	−	n.a.	n.a.	n.a.	−	−	−	+
Scoliosis/kyphosis	+	−	n.a.	+	n.a.	+	+ (mild scoliosis)	+ (severe scoliosis)	+
JHM at evaluation	−	n.a.	n.a.	n.a.	n.a.	+	−	−	−
Arachnodactyly/slender fingers	−	+	n.a.	n.a.	n.a.	+	−	−	+
Positional deformity feet	+ in infancy	−	n.a.	n.a.	n.a.	n.a.	−	−	−
Cubita/halluces/genua valga	−	n.a.	n.a.	+	n.a.	n.a.	−	−	n.a.
Minor body asymmetry	+	n.a.	n.a.	n.a.	n.a.	n.a.	−	−	n.a.
Other	Shortness of the III‐IV toe of the left foot	C4‐C5 fused vertebrae	Hyperlaxity, fractures and sprains				Dislocation of the hip	Sagittal craniosinostosis (surgery)	Mild contractures of elbows and knees
*Cardiovascular defects*									
Atrial/ventricular septal defects	Not reported	−	+	−	n.a.	−	+	+	−
Valvular defects	Not reported	−	−	+	n.a.	+	−	−	−
Arterial ectasia/dilatation (aortic bulb)	Not reported	n.a.	+	−	n.a.	−	−	−	n.a.
*Renal/urogenital anomalies*									
Vesicoureteral reflux	−	−	−	n.a.	n.a.	+	n.a.	−	−
Hydronephrosis	−	−	−	n.a.	n.a.	+	n.a.	−	−
Cryptorchidism/macrorchidism	−	n.a.	+	n.a.	n.a.	−	n.a.	−	+
*Ectodermal abnormalities*									
Multiple moles	−	n.a.	−	+	n.a.	+	+	−	n.a.
Hyper/hypopigmentation	−	n.a.	−	+	n.a.	+	−	−	+
Dry skin/eczema	−	n.a.	−	−	n.a.	n.a.	−	−	n.a.
Other ectodermal abnormalities	Small teeth		Elastic skin	Oligodontia, malocclusion, large central diastema, thick hair		Coarse and thick hair; absence of permanent lateral incisors		Alopecia	

	P10 (Koolen et al., 2016)	P11 (Nascimento et al., 2017)	P12 (Amenta et al., 2020)	P13 (Zollino et al., 2015)	P14 (Pascolini et al., 2021)	P15 (Koolen et al., 2006; Koolen et al., 2008)	P16 (Koolen et al., 2016)	P17 (Amenta et al., 2020)	P18 (Zollino et al., 2015)	P19 (Koolen et al., 2016)
Gender	Male	Female	Male	Male	Female	Male	Female	Male	Male	Female
Age, years	20	22	22	23	25	26	27	27	31	32
Genetics	17q21.31 microdeletion	17q21.31 microdeletion	17q21.31 microdeletion	17q21.31 microdeletion	17q21.31 microdeletion	17q21.31 microdeletion	17q21.31 microdeletion	17q21.31 microdeletion	17q21.31 microdeletion	17q21.31 microdeletion
Gestational age, weeks	40	42	40	n.a.	33 (PPROM)	term	42	40	n.a.	41
Birth weight, g	2700 (−2 *SD*)	2770	2880 (−1 *SD*)	3700	1370 (−1.88 *SD*)	3120	3950 (+0.5 *SD*)	2500 (−2 *SD*)	n.a.	2200 (−2.5 *SD*)
Height, cm	167 (−2.37 *SD*)	n.a.	161 (−2.5 *SD*)	156 (−3.5 *SD*)	146.5 cm (0 *SD*)	173 (10th centile)	165 (−0.92 *SD*)	166 (−2 *SD*)	n.a.	160 (−1.72 *SD*)
Postnatal short stature	+	n.a.	+	+	−	−	−	+	+	−
Weight, kg	n.a.	n.a.	44 (−3.5 *SD*)	39	53.5 (−1.88 *SD*)	68	66	104 (+2.8 *SD*)	n.a.	73
BMI	n.a.	n.a.	17	16.0	25	22.7	24.2	37.7	n.a.	28.5
*Neurological/neuropsychological features*										
Hypotonia	+	+	+	+	n.a.	++	+	+	+	+
Feeding problems	+	n.a.	−	−	+	+	+	−	−	+
Intellectual disability	+ severe	+	+ moderate	+ moderate	+ moderate	+ moderate	+ moderate/severe	+ mild	+ severe	+ moderate
Intelligence quotient (IQ)	n.a.	n.a.	45	n.a.	n.a.	40	<55	n.a.	n.a.	46
Seizures/EEG anomalies	+ Lennox Gastaut	+	+	+	+ (EEG)	−	+	−	+	+
Enlarged ventricles and hydrocephalus	+	n.a.	−	−	n.a.	+	+	−	−	n.a.
Other structural CNS anomalies	+ tetraplegia, atrofia cerebri	n.a.	+ partial agenesis of corpus callosum, nodular heterotopias	−	n.a.	n.a.	+ wide basal cisterna and cisterna magna, mild central and cortical atrophia	+ partial agenesis of corpus callosum, cerebellar vermis hypoplasia	−	−
Friendly/amiable affect	+	+	+	+	+	+	+	+	−	+
Stereotypic behavior	+	n.a.	−	+	−	n.a.	+	−	+	−
Anxiety	−	n.a.	−	−	+	n.a.	+	−	−	−
Difficulty to perform ADL	+	n.a.	+	−	−	n.a.	−	+	−	−
Other	Poor eye contact				Vomiting and anger crisis during air flight		Autism‐like symptoms			Psychosis
*Dysmorphic features*										
Long face	+	+	n.a.	+	+	+	+	+	+	+
Upslanting palpebral fissures	+	−	n.a.	+	+ (mildly)	−	+	−	−	+
Ptosis	−	−	n.a.	n.a.	−	+	−	−	−	+
Epicanthal folds	+	n.a.	n.a.	n.a.	−	−	+	+	−	−
Tubular or pear‐shaped nose	+	+	n.a.	+	+	+	+	+	+	+
Everted lower lip	+	+/−	n.a.	+	+	−	+	−	+	−
Large/prominent ears	+	+	n.a.	−	+	+	−	−	+	−
Other	Brachycephaly	Macrocephaly		Sparse eyebrow		Macrocephaly (63 cm), blepharophimosis, broad chin			Cleft palate	
*Hearing and visual impairment*										
Hypermetropia	−	n.a.	−	n.a.	−	−	+	−	n.a.	n.a.
Strabismus	+	n.a.	+	n.a.	−	+	+	−	+	+
Retinal impairment	−	n.a.	−	−	−	n.a.	+ left foveal scarring	−		−
Hearing impairment	+	n.a.	−	−	−	−	+	+	−	+
Other	Cerebral blindness				Nasal deviation and bilateral concha bullosa			Unilateral posterior cataract after birth		
*Musculoskeletal anomalies*										
Tracheo/laryngomalacia	−	n.a.	−	−	n.a.	n.a.	+	−	−	−
Pectus deformities	+	n.a.	−	−	−	−	−	−	−	−
Scoliosis/kyphosis	+	n.a.	+ (mild scoliosis)	+	+	+	+	−	+	+
JHM at evaluation	−	n.a.	+	+	−	+	+	−	−	+
Arachnodactyly/slender fingers	+	n.a.	−	−	+	+	−	−	−	−
Positional deformity feet	+	+	−	−	+ (flat feet)	+	+	+ (pes planus)	−	+
Cubita/halluces/genua valga	n.a.	n.a.	−	n.a.	+	+	n.a.	−	−	n.a.
Minor body asymmetry	−	n.a.	−	n.a.	n.a.	n.a.	+	−	−	+
Other	Contractures, small hands		Trigonocephaly (surgery)		Prognathism and malocclusion class III (surgery)					Hernia nuclei pulposi
*Cardiovascular defects*										
Atrial/ventricular septal defects	−	n.a.	−	−	+	−	−	−	−	−
Valvular defects	−	n.a.	−	–.	+	−	−	+ (bicuspid aortic)	−	−
Arterial ectasia/dilatation (aortic bulb)	−	n.a.	−	−	−	n.a.	−	+ (mild)	−	−
*Renal/urogenital anomalies*										
Vesicoureteral reflux	−	n.a.	+	−	−	−	−	−	−	−
Hydronephrosis	−	n.a.	−	+	−	−	−	−	−	−
Cryptorchidism/macrorchidism	+	n.a.	+	−	n.a.	+	−	+	+	−
*Ectodermal abnormalities*										
Multiple moles	−	n.a.	−	−	−	+	−	−	−	+
Hyper/hypopigmentation	−	n.a.	−	+	−	n.a.	−	−	−	−
Dry skin/eczema	−	n.a.	−	−	−	n.a.	+	−	n.a.	−
Other ectodermal abnormalities					Thin hairs; one speckled lentiginous nevus		Hypohidrosis		Abnormal hair/color texture	Small teeth

	P20 (Amenta et al., 2020)	P21 (Ciaccio et al., 2016)	P22 (Koolen et al., 2016)	P23 (Koolen et al., 2016)	P24 (Amenta et al., 2020)	P25 (Koolen et al., 2016)	P26 (Amenta et al., 2020; Zollino et al., 2015)	P27 (Koolen et al., 2016)	Total (when data available)
Gender	Male	Male	Male	Female	Female	Male	Female	Female	14 F and 13 M
Age, years	32	40	43	50	19	20	22	46	Range 18–63
Genetics	17q21.31 microdeletion	17q21.31 microdeletion	17q21.31 microdeletion	17q21.31 microdeletion	p.Pro667Leufs*3 in *KANSL1*	c.3125del; p.(Leu1042Argfs*71) in *KANSL1*	p.Arg929Glyfs*44 in *KANSL1*	c.908_909del; p.(Lys303Thrfs*11) in *KANSL1*	23 with 17q21.31 microdeletion and 4 with *KANSL1* pathogenic variant
Gestational age, weeks	40	44	40	46	40	40	41	40	
Birth weight, g	4360 (+3 *SD*)	2700 (−2 *SD*)	growth delay	3100 (−1.3 *SD*)	2900 (−1.5 *SD*)	3180 (−0.3 *SD*)	2850 (−1 *SD*)	3750 (+0.5 *SD*)	
Height, cm	175 (50th)	163 (−2.50 *SD*)	166.4 (−2.43 *SD*)	155 (−2.51 *SD*)	146 (−3 *SD*)	170.4 (−0.88 *SD*)	150 (−2.2 *SD*)	168 (−0.44 *SD*)	
Postnatal short stature	−	+	+	+	+	−	+	−	15/24 (63%)
Weight, kg	104 (+3 *SD*)	68	n.a.	52	59 (25th–50th)	n.a.	77 (90th)	n.a.	
BMI	33	26	n.a.	21.6	28	n.a.	32	n.a.	
*Neurological/neuropsychological features*									
Hypotonia	+	−	−	+	+	−	+	+	22/25 (88%)
Feeding problems	−	−	−	+	+	−	+	+	16/24 (67%)
Intellectual disability	+ mild	+ moderate	+ moderate	+ moderate	+ mild	+ mild	+ moderate	+ moderate	Moderate in 14/26 (54%)
Intelligence quotient (IQ)	58	48	35	n.a.	n.a.	61	42	n.a.	Range 35–61
Seizures/EEG anomalies	−	+ in infancy	−	−	−	+	−	−	16/26 (62%)
Enlarged ventricles and hydrocephalus	−	−	−	−	−	−	−	−	5/22 (23%)
Other structural CNS anomalies	−	−	+ rear developmental delay on the corpus callosum	−	−	−	−	+ dural ectasia and cysts	11/21 (52%)
Friendly/amiable affect	+	−	−	+	+	+	+	+	22/26 (85%)
Stereotypic behavior	−	−	−	+	−	+	−	−	7/21 (33%)
Anxiety	−	+	+	+	+	+	+	−	11/20 (55%)
Difficulty to perform ADL	−	+	−	−	−	+ severe	+	−	10/21 (48%)
Other		Poor eye contact		Depression episodes		Hypomimia	Panic attacks, depression	High pain threshold	
*Dysmorphic features*									
Long face	+	+	+	+	+	+	+	+	23/24 (96%)
Upslanting palpebral fissures	+	−	−	−	−	−	+	−	11/24 (46%)
Ptosis	−	−	+	−	+/−	−	n.a.	+	9/21 (43%)
Epicanthal folds	−	+	+	−	−	−	n.a.	−	7/20 (35%)
Tubular or pear‐shaped nose	+	+	+	+	+	−	+	+	24/25 (96%)
Everted lower lip	+/−	−	+	+	−	+	−	−	12/23 (52%)
Large/prominent ears	+	−	+	−	+	−	n.a.	−	10/24 (42%)
Other			Turricephaly		Thick eyebrows			Blepharophimosis	
*Hearing and visual impairment*									
Hypermetropia	−	−	+	−	−	−	−	+	4/20 (20%)
Strabismus	−	−	+	−	−	−	−	+	8/23 (35%)
Retinal impairment	−	+	−	−	−	−	−	−	3/21 (14%)
Hearing impairment	+	+	−	+	+	−	−	−	9/21 (43%)
Other	Bilateral cataract at birth		Optic nerve subatrophy						
*Musculoskeletal anomalies*									
Tracheo/laryngomalacia	−	−	−	−	−	−	+	−	3/18 (17%)
Pectus deformities	−	+ in infancy	−	−	−	−	−	−	3/23 (13%)
Scoliosis/kyphosis	−	+	+	+	+ (mild scoliosis)	−	+ (mild)	+	20/24 (83%)
JHM at evaluation	−	+	−	+	−	−	−	+	9/22 (41%)
Arachnodactyly/slender fingers	−	+	−	−	−	−	−	−	7/23 (30%)
Positional deformity feet	−	+	−	−	−	−	+	+	11/23 (48%)
Cubita/halluces/genua valga	−	+	n.a.	n.a.	−	n.a.	−	n.a.	4/13 (30%)
Minor body asymmetry	−	+	−	+	−	−	−	−	5/17 (29%)
Other	Sagittal craniosinostosis (surgery)	L5‐S1 spondylo‐listhesis	Large hallux	Spina bifida L2			Dislocation of the hip; sagittal craniosinostosis (surgery)		
*Cardiovascular defects*									
Atrial/ventricular septal defects	−	−	−	−	−	−	−	+	5/25 (20%)
Valvular defects	−	+ MVP	−	−	−	−	−	+	6/25 (24%)
Arterial ectasia/dilatation (aortic bulb)	−	+	−	−	−	−	−	−	3/22 (14%)
*Renal/urogenital anomalies*									
Vesicoureteral reflux	−	−	−	−	n.a.	−	−	−	2/22 (9%)
Hydronephrosis	−	−	−	−	n.a.	−	−	−	2/22 (9%)
Cryptorchidism/macrorchidism	+	−	+	−	n.a.	−	−	−	9/12 (75%) in males
*Ectodermal abnormalities*									
Multiple moles	−	−	−	+	−	−	−	+	7/23 (30%)
Hyper/hypopigmentation	−	−	+	+	−	−	−	−	6/23 (23%)
Dry skin/eczema	−	+	+	+	−	−	+	−	5/20 (25%)
Other ectodermal abnormalities			Vitiligo	Oligodontia			Oligodontia, abnormal hair/color texture	Oligodontia	

*Note*: Modified from Clinical Report of an Adult and Literature Review (Ciaccio et al., 2016) and integrated with other adult patients.

Abbreviations: ADL, activity of daily living; JHM, joint hypermobility; MVP, mitral valve prolapse; n.a., not available; PPROM, preterm premature rupture of membranes; *SD*, standard deviation.

^a^ She presents a de novo balanced translocation 46,XX,t(1;17)(q12;q21)dn disrupting *KANSL1* and a de novo microdeletion on the 16p11.2 atypical/distal region arr[hg18] 16q11.2 (28,732,295‐29,021,443)×1 dn.

The aunt was born post‐term from an assisted delivery with forceps. At birth, she suffered perinatal distress and during the first year of life, she had growth and feeding difficulties, which required hospitalization. She presented a delay of psychomotor development: she reached autonomous ambulation at the age of 2 years and the acquisition of language was delayed and characterized by stammering, which is still present. She attended special school and learned to read, write, and tell time, but she did not develop abstract mathematical reasoning and thinking. As a child, she had a few episodes of febrile convulsions, but no epilepsy occurred during her life. She has exhibited asthma since childhood, treated with corticosteroid therapy. The age at menarche was 14 years, then menses were regular. Standard karyotyping performed during adolescence was reported to be normal.

As an adult, ID is present and its degree is moderate. She has always been living with her family because she is not independent in her daily activities and she is now working for a special social cooperative. The behavioral phenotype is characterized by a friendly and sociable personality and her family reports a relatively strong memory for social‐contextual information, in particular for people close to her, such as family and friends. She has good conversational abilities, however with stammering; she learned to read and write as well as tell time. Nonetheless, numeracy is very difficult for her: she has not learned yet the four main calculations, she is still not able to handle money and did not develop abstract mathematical reasoning and thinking. She loves music very much and she sings at both home and work; she also likes attending theater courses in the special social cooperative she works in. In adulthood, she suffered by conductive hearing loss since her 40s and bilateral cataract was detected in her 50s and she reached menopause at 52 years. She presents scoliosis and a minor body asymmetry, but no cardiac, renal/urogenital, or skin abnormalities have ever been detected.

**FIGURE 1:**
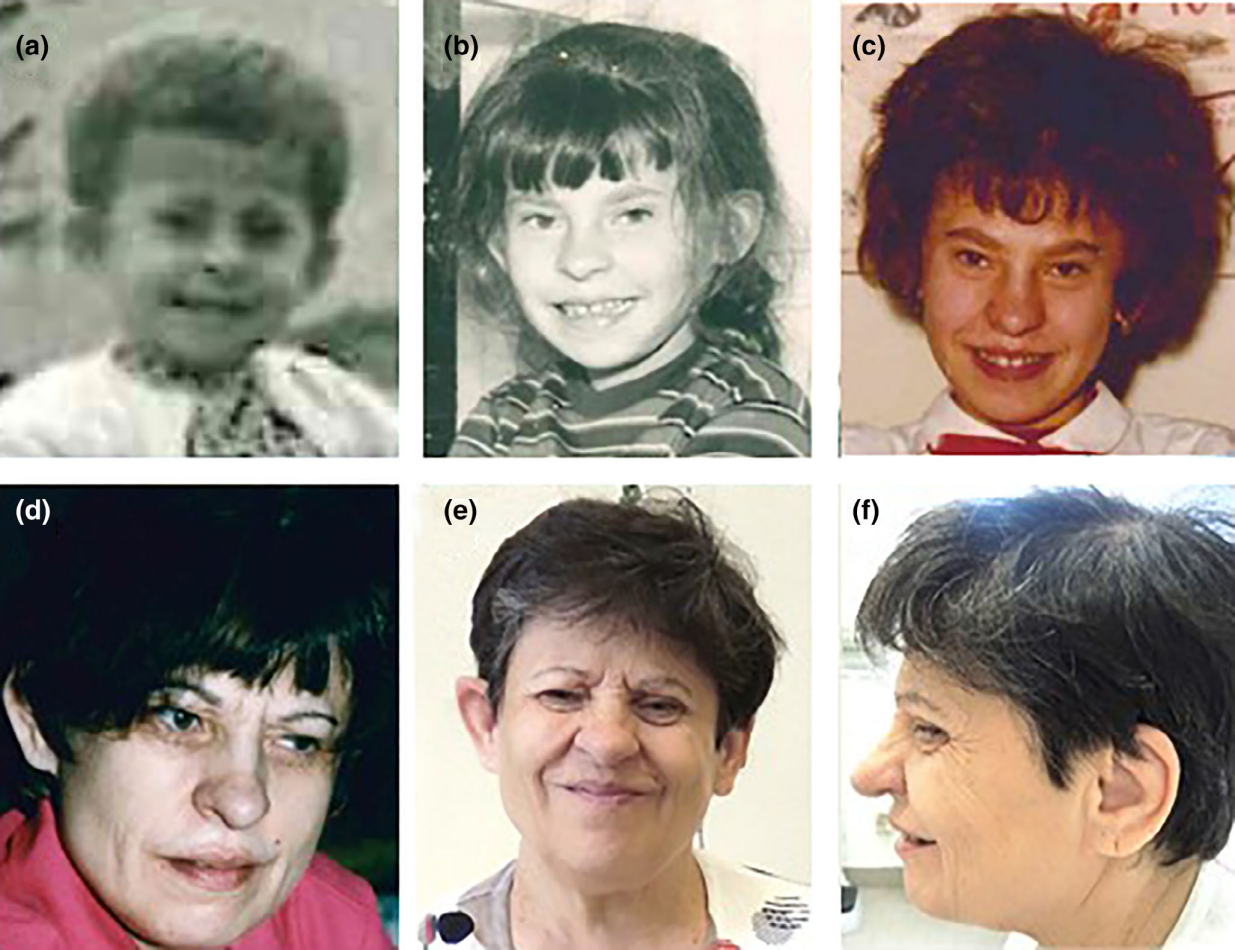
Photographs of our KdVS patient from infancy (a‐c) to adulthood (d‐f). (e and f) Show front and side profiles at last evaluation (62 years old): Narrow palpebral fissures and droopy eyelids became more evident as well as the broadening of the chin and a pronounced pear‐shaped nose. Notice in 1e a triangle‐shaped helix of the right ear, compared with the normal left ear in 1f

At her physical examination (Figure 1e,f), we noticed short stature (143 cm, <3rd percentile), span 144 cm, weight 60 kg (50th centile), macrocephaly (OCF 58 cm, >97th percentile), inner canthal distance 3.2 cm (mean), interpupillary distance 6.5 cm (97th centile), and outer canthal distance 9.8 cm (97th centile). Typical dysmorphism was observed: narrow palpebral fissures, droopy eyelids, pear‐shaped nose, bulbous overhanging nasal tip, broadening of the chin, large/prominent ears with a triangular‐shaped‐helix of the right ear, and shortness of the III‐IV toe of the left foot.

To better characterize the phenotype, we collected her photographs from infancy to now (Figure 1). As an infant (Figure 1a–c), she presented the typical facial dysmorphism of KdVS (upward slanting palpebral fissures, narrow/short palpebral fissures, large/prominent ears, and a pear‐shaped nose with a bulbous nasal tip). By observing the evolution of the facial phenotype of our patient during years (Figure 1d–f), it is possible to notice a coarsening of the dysmorphism, with broadening of the chin, a more pronounced pear‐shaped nose with bulbous overhanging nasal tip and more evident large/prominent ears.

### 2.2 Genetic analysis

Considering the differential diagnosis of syndromic ID (Miller et al., 2010), the aunt was investigated by array‐based Comparative Genomic Hybridization (aCGH) (180K; Agilent Technologies, Walldbronn, Germany), according to the manufacturer's protocol (v7.3; Agilent Technologies) and analyzed by CytoGenomics (v3.0.6.6; Agilent Technologies, ADM‐2 algorithm, release hg19). The copy number ratio of the DNA blood sample was compared to a female Human Genomic DNA (Agilent Technologies) as a reference. This analysis showed a microdeletion in the 17q21.31 region spanning about 504‐kb (chr17:43706886_44210822) establishing the diagnosis of KdVS (Figure S1A). This region encompassed eight genes of which two are OMIM (Online Mendelian Inheritance in Man) morbid genes: *MAPT* (*157140) and (partially) *KANSL1* (*612452), and three are OMIM: *CRHR1* (*122561)*, SPPL2C* (*608284), and *STH* (*607067) (uploaded into Decipher, 413465). Since her parents are deceased, familial segregation analysis was not performed.

In the pregnant niece, a karyotype analysis from peripheral blood resulted normal (46,XX) and after the KdVS diagnosis in her aunt was established, a FISH (Fluorescent In Situ Hybridization) analysis was carried out in order to exclude the presence of a balanced chromosomal rearrangement involving 17q21.31. This analysis with locus‐specific probe N0782E01, selected from the genomic library (32K Library; BACPAC Resources, Oakland, CA) according to Bernardini et al. (2007) was normal (Figure S1B). The pregnancy subsequently progressed uneventfully and ended with the birth of a healthy female.

## 3 REVIEW OF LITERATURE

### 3.1 Methods and subjects

After defining the diagnosis of our aunt's patient, we decided to search for all the adult patients with KdVS in literature. We searched for them on PubMed, by using “Koolen‐de Vries syndrome,” “17q21.31 microdeletion,” and “*KANSL1*” as keywords and selecting only patients who were at least 18‐year old at the time of clinical description. To the best of our knowledge, we identified 34 (with our patient included) adult patients, of whom 15 are males (14 with a 17q21.31 microdeletion, one with a *KANSL1* pathogenic variant) and 19 are females (16 with a 17q21.31 microdeletion, three with a *KANSL1* pathogenic variant) (Amenta et al., 2020; Ciaccio et al., 2016; Dubourg et al., 2011; Koolen et al., 2006, 2008, 2016; Moreno‐Igoa et al., 2015; Morgan et al., 2018; Myers et al., 2017; Nascimento et al., 2017; Pascolini et al., 2021; Shaw‐Smith et al., 2006; Terrone et al., 2012; Zollino et al., 2015). Since the clinical description of four adult patients in Myers et al. (2017) and three in Morgan et al. (2018) was more focused on epileptology and on speech development, respectively, we decided not to include these seven patients in the review (Table 1, totally 27 patients were included).

We evaluated both the photographs of adult patients, when available, and their clinical characteristics (Table 1) in order to compare our patient to the other adult individuals and to define the adult phenotype of the syndrome. Using the table available in the paper by Ciaccio et al. (2016) as a model, we verified every single patient and we added all the adult patients and their characteristics available from literature. The characteristics have been obtained from what declared in the papers (tables, text of the body, in some cases supplementary data) and, if dysmorphism were not declared, from patients' pictures, or If data could not be obtained, we opted for “n.a.” (not available). The percentage of each feature is calculated on data available. This adult case series includes 13 males and 14 females aged ≥18‐year old at the time of description and their age ranges from 18 to 63 years. Twenty‐three subjects present a 17q21.31 microdeletion and four present a heterozygous pathogenic *KANSL1* variant.

## 4 RESULTS

### 4.1 Auxological evaluation

Most adult patients (15/24, 63%) present a short stature. In females, the height ranges from 143 cm (−3 *SD*, our patient) to 168 cm; in males it ranges from 156 to 175 cm. While height seems to be decreased, weight tends to be increased in adult subjects. When the BMI (body mass index) was available (*n* = 14), overweight (25–29.9 kg/m^2^) or true obesity (≥ 30 kg/m^2^) occurred in eight (53%) of 15, while only two patients had a BMI below normal.

### 4.2 Neurological and behavioral features

Feeding problems and especially hypotonia were common problems during infancy (67% and 88%, respectively). As adults, ID is a constant feature and it is quite variable from mild to severe, but a moderate degree seems the most common (14/26, 54%). Seizure/EEG anomalies were present in the natural history of 62% of subjects, even if they seem to seizure‐free in adulthood. Structural CNS (central nervous system) anomalies are not uncommon, involving 52% of the subjects; they are variable but mainly involving enlargement of ventricules and dysgenesis/agenesis of corpus callosum.

From a behavioral standpoint, almost all adult patients (85%) present the distinctive friendly and amiable disposition but also anxiety is common (55%). Some experience autism‐like disorders, panic attacks, and depression. A difficulty to perform ADL (activity of daily living) is present in 48% of subjects.

### 4.3 Dysmorphic features

As adults, the long face and the tubular/pear‐shaped nose are almost invariably constant dysmorphic features (96%). Everted lower lip is present in 52% of patients. Eyes can be involved variably, with upslanting palpebral fissures (46%), ptosis (43%), and epicanthal folds (35%). Moreover, characteristic large/prominent ears may be present (42%); our patient presents a triangle‐shaped helix of the right ear. A broad chin is described in some adults. By observing photographs taken at various ages, when available, it is possible to confirm a coarsening of the dysmorphism, as they age.

### 4.4 Musculoskeletal anomalies

The musculoskeletal anomalies are quite common in adult patients with KdVS. The major characteristic is a spine abnormality, such as scoliosis/kyphosis (83%). Other typical features are the positional deformity of feet (48%) and the joint hypermobility (JHM), involving 41% of patients. Less common, but not rare, are arachnodactyly (30%), cubita/halluces/genua valga (30%), minor body asymmetry (29%), tracheo/larynghomalacia (17%), and pectus deformities (13%). Other musculoskeletal abnormalities have been less frequently described: craniosinostosis, prognathism and malocclusion class III, contractures, dislocation of the hip, shortness of the III‐IV toe of the left foot.

### 4.5 Hearing and visual impairment

A hearing involvement is not rare in KdVS: in particular, 43% of the adult patients show hearing impairment, either conductive or sensorineural. Eye involvement seems to be less frequent, but it is not uncommon: strabismus, hypermetropia, and retinal impairment are described in 35%, 20%, and 14%, respectively. In addition, cataract was reported in three patients, of whom two after birth and one in adulthood.

### 4.6 Cardiovascular and renal/urogenital defects

Heart defects can be present in adults, in particular valvular defects are described in 24% of subjects. Atrio‐ventricular septal defects are in 20% of adults and, less commonly, also arterial ectasia/dilatation, especially of aortic bulb, have been described (three patients).

Regarding urogenital defects, cryptorchidism/macrorchidism is a typical feature, reported in the natural history of 75% of adult male patients. Renal involvement is less frequent (vescicoureteral reflux and hydronephrosis both in 9%).

### 4.7 Ectodermal abnormalities

Anomalies involving skin, hair, and teeth are not uncommon. Multiple moles have been reported in seven (30%) of 23 adult patients, as well as hyper/hypopigmentation of the skin (6/23, 23%). Dry/skin eczema has been reported in five patients, oligodontia in five patients, and small teeth in two. A female young woman presents progressive alopecia from age 2 years.

## 5 DISCUSSION

The patient presented here is one of the rare adult subjects affected by KdVS; in particular, she seems to be the oldest affected individual reported in literature to the best of our knowledge (P1 in Table 1) and this patient provides additional information to the natural history of this rare condition.

It is noteworthy that we were able to collect her photographs from infancy to now. As a child (Figure 1a‐c), she presented upward slanting palpebral fissures, large/prominent ears and a pear‐shaped nose. As she aged (Figure 1d‐f), narrow palpebral fissures and droopy eyelids became more evident as well as a broadening of the chin and a pronounced pear‐shaped nose with bulbous overhanging nasal tip, which are described in the adult subjects (Koolen et al., 2008). In addition to that, ears became progressively larger. What is more, she also shows small teeth, a triangle‐shaped helix of the right ear, and an atypical shortness of the III‐IV toe of the left foot. This elf‐like feature together with her amiable behavior let us consider the Williams‐Beuren Syndrome (WBS) in the differential diagnosis (Egger et al., 2013). As an adult, our patient, who did not receive any specific treatment related to her disease apart from logopedics, showed moderate ID, mild speech impairment, kyphosis, conductive hearing loss since her 40s, bilateral cataracts detected in her 50s, in absence of other serious complications.

Comparing our patient to the second oldest patient known (P23 in Table 1, corresponding to patient 33, 50y, Supplementary 5 in Koolen et al., 2016, picture available in figure 2 of the same paper, patient T), our patient has a lower degree of autonomy than the other subject, but the main characteristics are shared. In particular, both have good conversational abilities; both can read and tell time and our patient can write simple sentences too. For both women, numeracy is very difficult, they are not able to handle money and did not develop abstract mathematical reasoning and thinking and our patient has not learned the four main calculations. Both women love music and theater very much.

In order to define an adult phenotype of KdVS, we searched for all the adult patients with KdVS in literature. We identified 34 (with ours included) adult individuals, of whom 15 are males (14 with a 17q21.31 microdeletion, one with a *KANSL1* pathogenic variant) and 19 are females (16 with a 17q21.31 microdeletion, three with a *KANSL1* pathogenic variant) (Amenta et al., 2020; Ciaccio et al., 2016; Dubourg et al., 2011; Koolen et al., 2006, 2008, 2016; Moreno‐Igoa et al., 2015; Morgan et al., 2018; Myers et al., 2017; Nascimento et al., 2017; Pascolini et al., 2021; Shaw‐Smith et al., 2006; Terrone et al., 2012; Zollino et al., 2015). We have summarized the main clinical characteristics of 27 adult patients, having a full clinical description (Table 1).

The case series include 13 males (12 with a 17q21.31 microdeletion and 1 with a *KANSL1* pathogenic variant) and 14 females (11 with a 17q21.31 microdeletion and 3 with a *KANSL1* pathogenic variant) aged ≥18 years, with ages that range from 18 to 63 years old. Few adult patients have been collected until now, because of the rarity of the disease and its relatively recent molecular definition: the aim of our review was to understand the natural history of this rare syndrome and to define its adult phenotype.

By observing their clinical data, some useful information about the adult phenotype of this rare syndrome can be deductable: first of all, most adults present short stature and a tendency to overweight/obesity, as suggested by (Amenta et al., 2020), even if in the majority of them, neonatal hypotonia and feeding difficulties in infancy were described. In particular, our patient, the oldest, has the shortest stature among them all (143 cm, −3 *SD*) and is overweight (BMI 29.3 kg/m^2^).

Concerning the neurological and behavioral features, we suggest they represent the distinctive elements of the disease in adulthood. Indeed, ID is a feature shared by all adult subjects, with a variable degree (usually moderate, 54% of adults) and it is typically accompanied by a specific behavioral pattern. Adults with KdVS show a typical friendly behavior, confirming this as a major sign both in children and in adults; it is interesting to point out that our patient has a strong memory for social information for people close to her, such as family and friends, characteristics that have been strongly reported to be present in KdVS (Egger et al., 2013). This over‐friendly disposition is often accompanied by behavior abnormalities, including anxiety (which seems to be quite common) and other possible disorders (panic attack, depression, shyness, etc…). Another important aspect to underline is that autonomy in daily activities often lacks or it is limited, as well as language skills, which may be poor in some adults (Amenta et al., 2020; Morgan et al., 2018).

In addition, in the collected adult subjects, a history of seizures is described in 62% of cases, which is slightly more than what is known in literature (Myers et al., 2017). Interestingly, seizures tend to remission and to be absent in adulthood; therefore, a detailed personal history should be carefully recorded, to avoid missing details in childhood (Amenta et al., 2020). Our review shows that also structural CNS abnormalities are variably reported in 52% of adults, as expected from literature, and are universal (Myers et al., 2017) even if enlargement of ventricles and dysgenesis/agenesis of corpus callosum seem to be the most frequent MRI findings.

Regarding dysmorphic features, almost all adult patients collected share a long face and a pronounced pear‐shaped nose with bulbous overhanging nasal tip (Koolen et al., 2008): according to us, these two elements together should evoke the suspect of KdVS both in childhood and in adulthood. Moreover, by observing photographs taken at various ages, it is possible to confirm a widening of the chin and a coarsening of the dysmorphism as they age (Koolen et al., 2008). What is more, peculiar but not constant aspects of KdVS in adulthood are the presence of everted lower lip and large/prominent ears: when present, these features may represent a confounding factor because they may evoke, together with the friendly behavior, a diagnosis of Williams‐Beuren syndrome in adulthood. This consideration does not apply to childhood when the facial gestalt of WBS is instead distinctive (Kruszka et al., 2018).

Concerning other body districts, this review suggests that the major congenital abnormalities present in adulthood are the musculoskeletal findings, which are quite common in adult patients with KdVS. We detected, in particular, spine (kyphosis/scoliosis) and feet deformities and joint hypermobility (JHM) but many other defects have been reported. The spine deformities may justify the presence of short stature in adulthood while feet deformity may be an early sign of the disease. For example, we identified in our routine clinic activity a prenatal case of KdVS by array‐CGH on DNA extracted from amniotic fluid in a male fetus that presented with bilateral clubfoot, a cardiac hyperechoic focus, a malformation of the urinary tract, and a high‐risk screening test for trisomy 13 and 18.

Regarding other characteristics, data from male adult patients suggest a history of crypto/macrorchidism is often present and data from all patients show that hearing impairment, either conductive or sensorineural is not uncommon, involving about half of the subjects. It is also necessary to consider the presence of ocular, cardiovascular (especially involving valvules), and renal defects that seem to be variably present but not to be distinctive features of the disease.

Finally, it is noteworthy that ectodermal abnormalities may represent a peculiar additional aspect of the disease; therefore, anomalies involving skin, hair, and teeth (oligodontia included) should be fully investigated during clinical evaluation.

To sum up, from an overall analysis of literature concerning KdVS and adult subjects, it emerged that it is possible to discriminate the phenotype in the different stages of life. In particular, it seems that childhood is characterized by hypotonia and feeding difficulties in the majority of children and by epilepsy in about 50% (Koolen et al., 2008). With increasing age, we noticed a progressive stability of the clinical picture, characterized by moderate ID, non‐evolution of the neurobehavioral disorders, recovery from epilepsy, and absence of major internal organ involvement (Amenta et al., 2020). As for facial dysmorphism, they are present since birth and they tend to remain stable over time, although in childhood there is a tendency to hypotonic face, with open mouth appearance and protruding tongue, while in adulthood there is a tendency to elongation of the face, broadening of the chin and to a more pronounced tubular/pear‐shaped nose (Amenta et al., 2020; Koolen et al., 2008).

To conclude, we propose that the cardinal features of KdVS in adulthood are ID (ranging from mild to severe, but usually moderate), friendly behavior, musculoskeletal abnormalities (especially scoliosis, JHM and feet deformity), and facial dysmorphism (a long face and a pronounced pear‐shaped nose with bulbous nasal tip). Therefore, we suggest considering KdVS in differential diagnosis in adult patients characterized by these features, by opting for firstly an array‐CGH analysis to search for 17q21.31 microdeletion and, if negative, for a *KANSL1* sequencing to detect heterozygous pathogenic variants.

As more and more young and adult individuals are identified, further studies are necessary to define the long‐term prognosis of patients with KdVS and to what extent supporting treatments can improve their phenotype.

## CONFLICT OF INTEREST

The authors have no conflicts of interest to declare.

## AUTHOR CONTRIBUTIONS

Marianna Farnè searched for literature and wrote the manuscript with major support from Stefania Bigoni, who supervised the project. Mariabeatrice Sanchini elaborated data. Laura Bernardini, Anna Capalbo, Mariabeatrice Sanchini, Sergio Fini, Giusy Cavarretta, Barbara Torres, and Alessandra Ferlini contributed to the revision of the final manuscript. Laura Bernardini, Anna Capalbo, Barbara Torres, Giusy Cavarretta, and Sergio Fini carried out the experiments.

## Supporting information


**Figure S1**. (A) Genomic profile of Chromosome 17 and the detailed view of the microdeletion of about 504 ‐kb in 17q21.31; (B) FISH analysis performed on the pregnant niece with N0782E01 (orange) shows a normal hybridization pattern on the Chromosomes 17.

## Data Availability

The data that support the findings of this study are available from the corresponding author upon reasonable request.

## References

[ajmga62536-bib-0001] Amenta, S. , Frangella, S. , Marangi, G. , Lattante, S. , Ricciardi, S. , Doronzio, P. N. , Orteschi, D. , Veredice, C. , Contaldo, I. , Zampino, G. , Gentile, M. , Scarano, E. , Graziano, C. , & Zollino, M. (2020). Adult phenotype in Koolen‐de Vries/KANSL1 haploinsufficiency syndrome. Journal of Medical Genetics, jmedgenet‐2020‐107225. 10.1136/jmedgenet-2020-107225 Epub ahead of print. 33361104

[ajmga62536-bib-0002] Bernardini, L. , Capalbo, A. , D'Avanzo, M. G. , Torrente, I. , Grammatico, P. , Dell'Edera, D. , Cavalcanti, D. P. , Novelli, A. , & Dallapiccola, B. (2007). Five cases of supernumerary small ring chromosomes 1: Heterogeneity and genotype‐phenotype correlation. European Journal of Medical Genetics, 50(2), 94–102. 10.1016/j.ejmg.2006.11.001 Epub 2006 Nov 23. 17236832

[ajmga62536-bib-0003] Ciaccio, C. , Dordoni, C. , Ritelli, M. , & Colombi, M. (2016). Koolen‐de Vries syndrome: Clinical report of an adult and literature review. Cytogenetic and Genome Research, 150(1), 40–45. 10.1159/000452724. Epub 2016 Nov 17 Decipher, https://decipher.sanger.ac.uk/patient/413465 27852077

[ajmga62536-bib-0004] Dubourg, C. , Sanlaville, D. , Doco‐Fenzy, M. , Le Caignec, C. , Missirian, C. , Jaillard, S. , Schluth‐Bolard, C. , Landais, E. , Boute, O. , Philip, N. , Toutain, A. , David, A. , Edery, P. , Moncla, A. , Martin‐Coignard, D. , Vincent‐Delorme, C. , Mortemousque, I. , Duban‐Bedu, B. , Drunat, S. , … Andrieux, J. (2011). Clinical and molecular characterization of 17q21.31 microdeletion syndrome in 14 French patients with mental retardation. European Journal of Medical Genetics, 54(2), 144–151. 10.1016/j.ejmg.2010.11.003 Epub 2010 Nov 20. 21094706

[ajmga62536-bib-0005] Egger, J. I. , Wingbermühle, E. , Verhoeven, W. M. , Dijkman, M. , Radke, S. , de Bruijn, E. R. , de Vries, B. , Kessels, R. P. , & Koolen, D. (2013). Hypersociability in the behavioral phenotype of 17q21.31 microdeletion syndrome. American Journal of Medical Genetics. Part A, 161A(1), 21–26. 10.1002/ajmg.a.35652 Epub 2012 Nov 20. 23169757

[ajmga62536-bib-0006] Koolen, D. A. , Dupont, J. , de Leeuw, N. , Vissers, L. E. , van den Heuvel, S. P. , Bradbury, A. , Steer, J. , de Brouwer, A. P. , Ten Kate, L. P. , Nillesen, W. M. , de Vries, B. B. , & Parker, M. J. (2012). Two families with sibling recurrence of the 17q21.31 microdeletion syndrome due to low‐grade mosaicism. European Journal of Human Genetics, 20(7), 729–733. 10.1038/ejhg.2012.1 Epub 2012 Feb 1. 22293690 PMC3376266

[ajmga62536-bib-0007] Koolen, D. A. , Kramer, J. M. , Neveling, K. , Nillesen, W. M. , Moore‐Barton, H. L. , Elmslie, F. V. , Toutain, A. , Amiel, J. , Malan, V. , Tsai, A. C. , Cheung, S. W. , Gilissen, C. , Verwiel, E. T. , Martens, S. , Feuth, T. , Bongers, E. M. , de Vries, P. , Scheffer, H. , Vissers, L. E. , … de Vries, B. B. (2012). Mutations in the chromatin modifier gene KANSL1 cause the 17q21.31 microdeletion syndrome. Nature Genetics, 44(6), 639–641. 10.1038/ng.2262 22544363

[ajmga62536-bib-0008] Koolen, D. A. , Pfundt, R. , Linda, K. , Beunders, G. , Veenstra‐Knol, H. E. , Conta, J. H. , Fortuna, A. M. , Gillessen‐Kaesbach, G. , Dugan, S. , Halbach, S. , Abdul‐Rahman, O. A. , Winesett, H. M. , Chung, W. K. , Dalton, M. , Dimova, P. S. , Mattina, T. , Prescott, K. , Zhang, H. Z. , Saal, H. M. , … de Vries, B. B. (2016). The Koolen‐de Vries syndrome: A phenotypic comparison of patients with a 17q21.31 microdeletion versus a KANSL1 sequence variant. European Journal of Human Genetics, 24(5), 652–659. 10.1038/ejhg.2015.178 Epub 2015 Aug 26. 26306646 PMC4930086

[ajmga62536-bib-0009] Koolen, D. A. , Sharp, A. J. , Hurst, J. A. , Firth, H. V. , Knight, S. J. , Goldenberg, A. , Saugier‐Veber, P. , Pfundt, R. , Vissers, L. E. , Destrée, A. , Grisart, B. , Rooms, L. , Van der Aa, N. , Field, M. , Hackett, A. , Bell, K. , Nowaczyk, M. J. , Mancini, G. M. , Poddighe, P. J. , … de Vries, B. B. (2008). Clinical and molecular delineation of the 17q21.31 microdeletion syndrome. Journal of Medical Genetics, 45(11), 710–720. 10.1136/jmg.2008.058701 Epub 2008 Jul 15. Erratum in: J Med Genet. 2009 Aug;46(8):576. 18628315 PMC3071570

[ajmga62536-bib-0010] Koolen, D. A. , Vissers, L. E. , Pfundt, R. , de Leeuw, N. , Knight, S. J. , Regan, R. , Kooy, R. F. , Reyniers, E. , Romano, C. , Fichera, M. , Schinzel, A. , Baumer, A. , Anderlid, B. M. , Schoumans, J. , Knoers, N. V. , van Kessel, A. G. , Sistermans, E. A. , Veltman, J. A. , Brunner, H. G. , & de Vries, B. B. (2006). A new chromosome 17q21.31 microdeletion syndrome associated with a common inversion polymorphism. Nature Genetics, 38(9), 999–1001. 10.1038/ng1853 Epub 2006 Aug 13. 16906164

[ajmga62536-bib-0011] Kruszka, P. , Porras, A. R. , de Souza, D. H. , Moresco, A. , Huckstadt, V. , Gill, A. D. , Boyle, A. P. , Hu, T. , Addissie, Y. A. , Mok, G. T. K. , Tekendo‐Ngongang, C. , Fieggen, K. , Prijoles, E. J. , Tanpaiboon, P. , Honey, E. , Luk, H. M. , Lo, I. F. M. , Thong, M. K. , Muthukumarasamy, P. , … Muenke, M. (2018). Williams‐Beuren syndrome in diverse populations. American Journal of Medical Genetics. Part A, 176(5), 1128–1136. 10.1002/ajmg.a.38672 29681090 PMC6007881

[ajmga62536-bib-0012] Miller, D. T. , Adam, M. P. , Aradhya, S. , Biesecker, L. G. , Brothman, A. R. , Carter, N. P. , Church, D. M. , Crolla, J. A. , Eichler, E. E. , Epstein, C. J. , Faucett, W. A. , Feuk, L. , Friedman, J. M. , Hamosh, A. , Jackson, L. , Kaminsky, E. B. , Kok, K. , Krantz, I. D. , Kuhn, R. M. , … Ledbetter, D. H. (2010). Consensus statement: Chromosomal microarray is a first‐tier clinical diagnostic test for individuals with developmental disabilities or congenital anomalies. American Journal of Human Genetics, 86(5), 749–764. 10.1016/j.ajhg.2010.04.006 20466091 PMC2869000

[ajmga62536-bib-0013] Moreno‐Igoa, M. , Hernández‐Charro, B. , Bengoa‐Alonso, A. , Pérez‐Juana‐del‐Casal, A. , Romero‐Ibarra, C. , Nieva‐Echebarria, B. , & Ramos‐Arroyo, M. A. (2015). KANSL1 gene disruption associated with the full clinical spectrum of 17q21.31 microdeletion syndrome. BMC Medical Genetics, 16, 68. 10.1186/s12881-015-0211-0 26293599 PMC4593202

[ajmga62536-bib-0014] Morgan, A. T. , Haaften, L. V. , van Hulst, K. , Edley, C. , Mei, C. , Tan, T. Y. , Amor, D. , Fisher, S. E. , & Koolen, D. A. (2018). Early speech development in Koolen de Vries syndrome limited by oral praxis and hypotonia. European Journal of Human Genetics, 26(1), 75–84. 10.1038/s41431-017-0035-9 Epub 2017 Dec 11. 29225339 PMC5839037

[ajmga62536-bib-0015] Myers, K. A. , Mandelstam, S. A. , Ramantani, G. , Rushing, E. J. , de Vries, B. B. , Koolen, D. A. , & Scheffer, I. E. (2017). The epileptology of Koolen‐de Vries syndrome: Electro‐clinico‐radiologic findings in 31 patients. Epilepsia, 58(6), 1085–1094. 10.1111/epi.13746 28440867

[ajmga62536-bib-0016] Nascimento, G. R. , Pinto, I. P. , de Melo, A. V. , da Cruz, D. M. , Ribeiro, C. L. , da Silva, C. C. , da Cruz, A. D. , & Minasi, L. B. (2017). Molecular characterization of Koolen De Vries syndrome in two girls with idiopathic intellectual disability from Central Brazil. Molecular Syndromology, 8(3), 155–160. 10.1159/000456910 Epub 2017 Feb 24. 28588437 PMC5448449

[ajmga62536-bib-0017] Pascolini, G. , Gaudioso, F. , Fadda, M. T. , Laino, L. , Ferraris, A. , & Grammatico, P. (2021). Koolen‐de Vries syndrome in the first adulthood patient of southern India ancestry. American Journal of Medical Genetics. Part A, 185(3), 978–981. 10.1002/ajmg.a.62006 Epub 2020 Dec 12. 33314579

[ajmga62536-bib-0018] Sauvestre, F. , Marguet, F. , Rooryck, C. , Vuillaume, M. L. , Cardinaud, F. , Laquerrière, A. , André, G. , & Pelluard, F. (2017). Early fetal presentation of Koolen‐de Vries: Case report with literature review. European Journal of Medical Genetics, 60(11), 605–609. 10.1016/j.ejmg.2017.08.012 Epub 2017 Aug 12. 28811189

[ajmga62536-bib-0019] Sharp, A. J. , Hansen, S. , Selzer, R. R. , Cheng, Z. , Regan, R. , Hurst, J. A. , Stewart, H. , Price, S. M. , Blair, E. , Hennekam, R. C. , Fitzpatrick, C. A. , Segraves, R. , Richmond, T. A. , Guiver, C. , Albertson, D. G. , Pinkel, D. , Eis, P. S. , Schwartz, S. , Knight, S. J. , & Eichler, E. E. (2006). Discovery of previously unidentified genomic disorders from the duplication architecture of the human genome. Nature Genetics, 38(9), 1038–1042. 10.1038/ng1862 Epub 2006 Aug 13. 16906162

[ajmga62536-bib-0020] Shaw‐Smith, C. , Pittman, A. M. , Willatt, L. , Martin, H. , Rickman, L. , Gribble, S. , Curley, R. , Cumming, S. , Dunn, C. , Kalaitzopoulos, D. , Porter, K. , Prigmore, E. , Krepischi‐Santos, A. C. , Varela, M. C. , Koiffmann, C. P. , Lees, A. J. , Rosenberg, C. , Firth, H. V. , de Silva, R. , & Carter, N. P. (2006). Microdeletion encompassing MAPT at chromosome 17q21.3 is associated with developmental delay and learning disability. Nature Genetics, 38(9), 1032–1037. 10.1038/ng1858 Epub 2006 Aug 13. 16906163

[ajmga62536-bib-0021] Terrone, G. , D'Amico, A. , Imperati, F. , Carella, M. , Palumbo, O. , Gentile, M. , Canani, R. B. , Melis, D. , Romano, A. , Parente, I. , Riccitelli, M. , & Del Giudice, E. (2012). A further contribution to the delineation of the 17q21.31 microdeletion syndrome: Central nervous involvement in two Italian patients. European Journal of Medical Genetics, 55(8–9), 466–471. 10.1016/j.ejmg.2012.04.010 22659270

[ajmga62536-bib-0022] Zollino, M. , Marangi, G. , Ponzi, E. , Orteschi, D. , Ricciardi, S. , Lattante, S. , Murdolo, M. , Battaglia, D. , Contaldo, I. , Mercuri, E. , Stefanini, M. C. , Caumes, R. , Edery, P. , Rossi, M. , Piccione, M. , Corsello, G. , Della Monica, M. , Scarano, F. , Priolo, M. , … Zackai, E. (2015). Intragenic KANSL1 mutations and chromosome 17q21.31 deletions: Broadening the clinical spectrum and genotype‐phenotype correlations in a large cohort of patients. Journal of Medical Genetics, 52(12), 804–814. 10.1136/jmedgenet-2015-103184 Epub 2015 Sep 30. 26424144

[ajmga62536-bib-0023] Zollino, M. , Orteschi, D. , Murdolo, M. , Lattante, S. , Battaglia, D. , Stefanini, C. , Mercuri, E. , Chiurazzi, P. , Neri, G. , & Marangi, G. (2012). Mutations in KANSL1 cause the 17q21.31 microdeletion syndrome phenotype. Nature Genetics, 44(6), 636–638. 10.1038/ng.2257 22544367

