# Supplementary figures and images for: Koolen‐de Vries syndrome in a 63‐year‐old woman: Report of the oldest patient and a review of the adult phenotype
Source: Am J Med Genet A. 2021 Oct 19;188(2):692–707. doi: 10.1002/ajmg.a.62536 (PMC9297928; doi:10.1002/ajmg.a.62536)

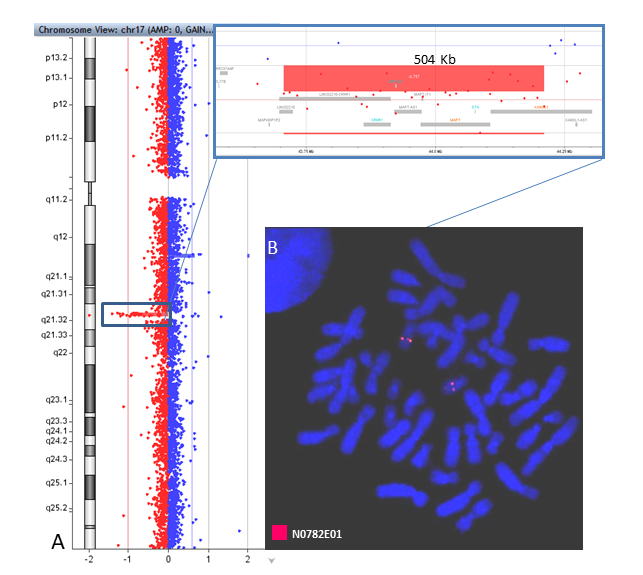

Supplement: Supplementary file 1 — Figure S1. (A) Genomic profile of Chromosome 17 and the detailed view of the microdeletion of about 504 ‐kb in 17q21.31; (B) FISH analysis performed on the pregnant niece with N0782E01 (orange) shows a normal hybridization pattern on the Chromosomes 17. [file AJMG-188-692-s001.tif]
